# Infectious hepatitis E virus is associated with the mature sperm head

**DOI:** 10.1371/journal.ppat.1012240

**Published:** 2024-05-20

**Authors:** Kush K. Yadav, Patricia A. Boley, Thamonpan Laocharoensuk, Saroj Khatiwada, Carolyn M. Lee, Menuka Bhandari, Lindsey Moore, Juliette Hanson, Scott P. Kenney

**Affiliations:** 1 Center for Food Animal Health, Department of Animal Sciences, The Ohio State University, Wooster, Ohio, United States of America; 2 Department of Veterinary Preventive Medicine, The Ohio State University, College of Veterinary Medicine, Columbus, Ohio, United States of America; 3 The College of Wooster, Wooster, Ohio, United States of America; 4 Plant and Animal Agrosecurity Research Facility, Center for Food Animal Health, Department of Animal Sciences, The Ohio State University, Wooster, Ohio, United States of America; Tsinghua University, CHINA

## Abstract

Hepatitis E virus (HEV) is the leading cause of acute viral hepatitis worldwide. HEV associated pregnancy mortality has been reported as up to 30% in humans. Recent findings suggest HEV may elicit effects directly in the reproductive system with HEV protein found in the testis, viral RNA in semen, and viral replication occurring in placental cell types. Using a natural host model for HEV infection, pigs, we demonstrate infectious HEV within the mature spermatozoa and altered sperm viability from HEV infected pigs. HEV isolated from sperm remained infectious suggesting a potential transmission route via sexual partners. Our findings suggest that HEV should be explored as a possible sexually transmittable disease. Our findings propose that infection routes outside of oral and intravenous infection need to be considered for their potential to contribute to higher mortality in HEV infections when pregnancy is involved and in HEV disease in general.

## Author summary

Hepatitis E virus (HEV) is one of the major causes of acute viral hepatitis worldwide. Extrahepatic manifestations have been described for HEV including neurological, pancreatic, and reproductive disorders. Recent findings have suggested that HEV can be found in the testes, is associated with male infertility, and viral RNA can be detected in semen. HEV can replicate in human testicular explants and Sertoli cells, and replicates in the vaginal tissue of rabbits. We demonstrate that infectious HEV is associated with the sperm head. The infectious ability of HEV present in sperm suggests a potentially new transmission route of HEV that necessitates in-depth study. Our study adds to the existing literature highlighting the importance of understanding HEV association with sperm as a potential contributor as a sexually transmittable disease. New studies to evaluate sexual transmission of HEV, its association with pregnancy mortality, and the testes as a potential virus reservoir are necessary.

## Introduction

Hepatitis E has been increasingly recognized as an emerging and significant clinical problem in immunocompromised and pregnant humans globally [1]. HEV infection is the leading cause of acute viral hepatitis worldwide causing significant liver damage [2], eventually leading to cirrhosis [3] in some cases, with considerable mortality in humans [4]. A recent report ranked hepatitis E virus (HEV) 6^th^ out of 887 wildlife viruses with significant risk of spilling over into humans, behind only Lassa, SARS-CoV-2, Ebola, Hanta, and Nipah viruses, but ahead of viral pathogens such as monkeypox, Marburg, rabies, and other emerging coronaviruses [5].

Recent reports have found the presence of HEV RNA in semen at a higher incidence rate in infertile men [6]. HEV RNA has also been detected within semen in chronic hepatitis E [7] patients, but infectious virus could not be cultured in either instance. Approximately 50% of clinical male infertility cases are due to unknown causes [8]. It is currently unknown whether some of these infertility cases could be linked to asymptomatic HEV infection. Human and animal studies (nonhuman primates, gerbils, mice, and pigs) detected HEV RNA in semen and protein in the testis associated with alteration in sperm motility and morphology [6,9–11]. Detection of HEV within the testis could simply be due to local inflammation compromising the blood testis barrier resulting in virus entry and replication at a normally immune privileged site (6). Even though HEV RNA has been detected in human semen and immature spermatogonia, the relationship of the virus with mature spermatozoa is unclear with current evidence lacking for the presence of the virus in the sperm cell itself [6].

We used a natural host, the pig, infected with the genotype 3 zoonotic strain of *Paslahepevirus balayani* HEV (US-2 isolate) as a model to investigate the presence of HEV in sperm cells. We further assessed the infectiousness of virus within sperm cells using a human liver cell culture system to understand the potential transmission ability of HEV via sperm cells.

## Results

### Active sub-clinical HEV infection in pigs

**Fig 1:**
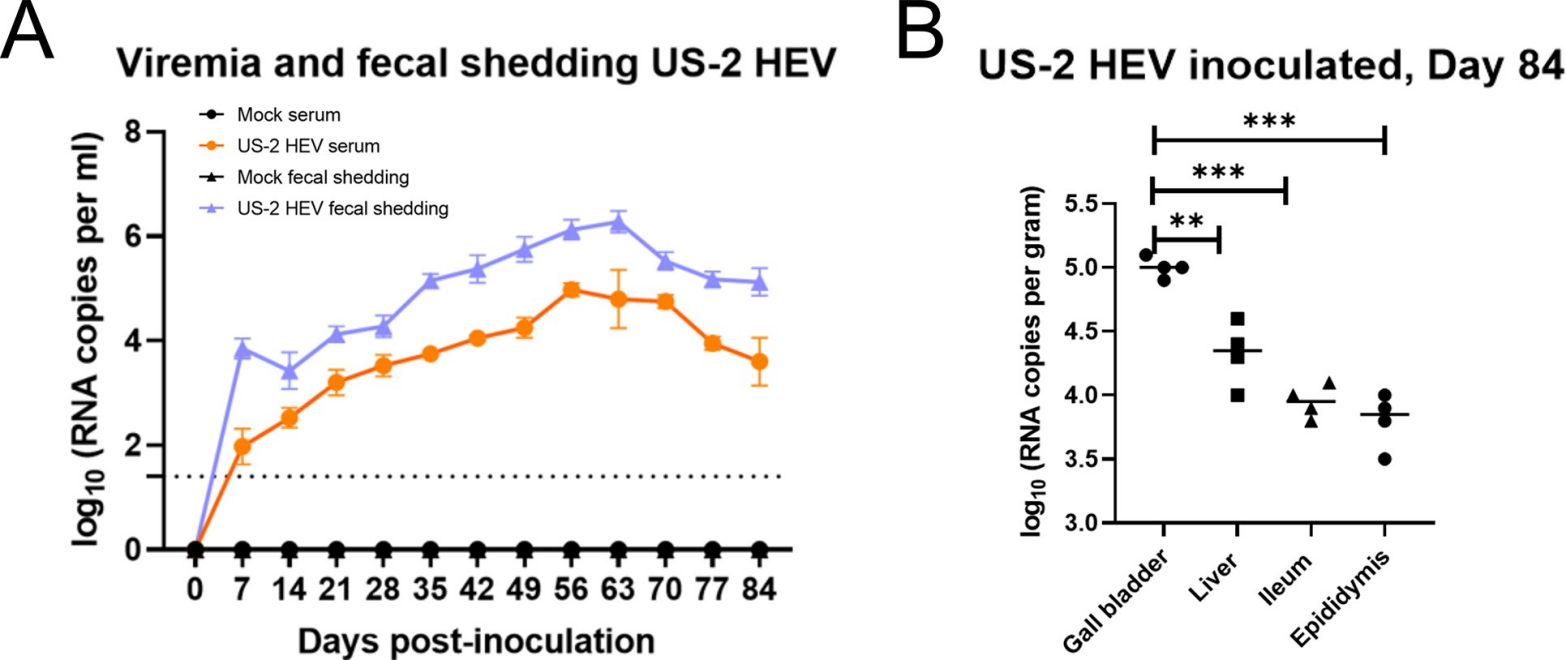
Active sub-clinal infection in pigs. (A) Viremia and fecal viral shedding from pigs inoculated with US-2 HEV. Weekly RNA data are presented. Mock-infected groups remained negative throughout the study. (B) US-2 HEV tissue distribution at 84 days post-inoculation. HEV RNA loads in gall bladder, liver, ileum, and epididymis from US-2 HEV inoculated pigs. Negative control pigs used in the study remained negative. ** = p < 0.01, *** = p < 0.001.

In this study, we utilized a natural HEV host with similar human size and male reproductive physiology, the pig. Viremia, fecal viral shedding, higher RNA titers in the gall bladder, liver, ileum, and epididymis in US-2 HEV inoculated pigs was observed until day 84 and demonstrated active sub-clinical HEV infection (Fig 1A and 1B). Although viremia and fecal viral shedding was seen as early as day 7 in the US-2 HEV inoculated group, it could be the remnants of the inoculum. Replication kinetics studies showed a clear increase in the viral RNA in serum and feces over the course of infection.

### Presence of HEV in the sperm head

**Fig 2:**
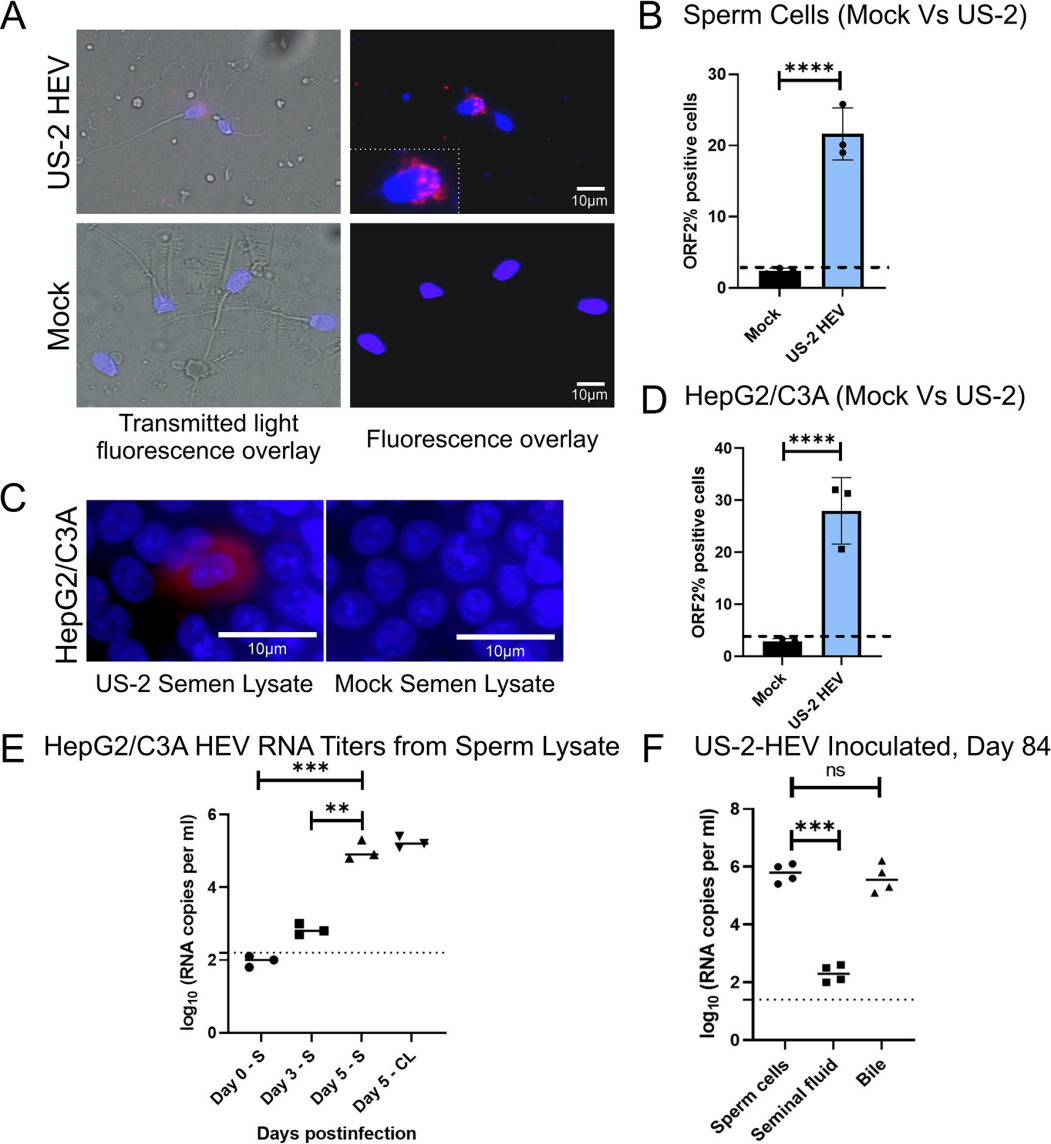
Hepatitis E virus infects spermatozoa. (A) Immunohistochemical detection of hepatitis E (red) in the head of spermatozoa obtained from the US-2 infected pigs at day 84 post infection; Hepatitis E virus ORF2 is red; DAPI stain is blue (nucleus). (B) Flow cytometry analysis demonstrating the percentage of sperm cells infected with hepatitis E virus (US-2 strain). (C) Immunodetection of hepatitis E demonstrating infectious sperm cells in HepG2/C3A cells. (D) Flow cytometry analysis demonstrating the percentage of HepG2/C3A cells infected with the sperm cell lysates from mock and US-2 HEV infected pigs. (E) Infectious titer of sperm-derived HEV using HepG2/C3A cells. US-2 HEV RNA loads in culture supernatant (S) and cell lysates (CL) of HepG2/C3A cell cultures after inoculation with the lysed sperm cells. Independent biological experiments, mean ± SD of three replicates, are presented. The dotted line represents the cut-off value demonstrating the background referring to the attachments of the virus to the cell surfaces. (F) HEV RNA loads in sperm cells suspension, seminal fluid, and bile from US-2 HEV inoculated pigs. *** = p < 0.001, **** = p < 0.0001.

We report the immunohistochemical detection of HEV in the head of spermatozoa obtained at 84 days post intravenous inoculation of conventional pigs (Fig 2A). The use of fluorescence microscopy demonstrated HEV ORF2 antigen within the acrosomal region of the sperm head, while non-infected mock group sperm showed no HEV staining, confirming the specificity of the assay. Using flow cytometry analysis, we were able to demonstrate that at least 19% of the sperm cells contained HEV antigens (Fig 2B).

### Lysates from sperm cells collected from US-2 HEV infected pigs contain virus infectious to HepG2/C3A cells

To study whether the HEV antigen detected in the sperm cells represented infectious virus, we lysed sperm cells and inoculated ~4.8 x 10^5^ viral RNA copies onto human liver cells (HepG2/C3A) *in-vitro*. We assessed events at the single cell level using indirect immunofluorescence employing antibodies directed against ORF2 capsid protein. Immunofluorescence detection of HEV ORF2 in HepG2/C3A cells five days post inoculation with sperm cell lysates demonstrates HEV within the sperm cells remains infectious (Fig 2C). In addition, flow cytometry quantification of HepG2/C3A cells demonstrates at least 23% of cells contained HEV antigens (Fig 2D). HEV replication ability was further demonstrated by an increase in the viral RNA copies in the HepG2/C3A cellular lysate over time and in the supernatant harvested from inoculated HepG2/C3A cells (Fig 2E). Higher HEV RNA copies were detected in sperm cells lysates in comparison to the seminal fluid (Fig 2F) but almost equivalent to the bile (Fig 2E). Interestingly, sperm cell viral RNA load was higher than seen in the gall bladder, liver, ileum, and epididymis (Fig 1B)

### HEV infection status correlates with altered motility and morphology of sperm cells

**Fig 3:**
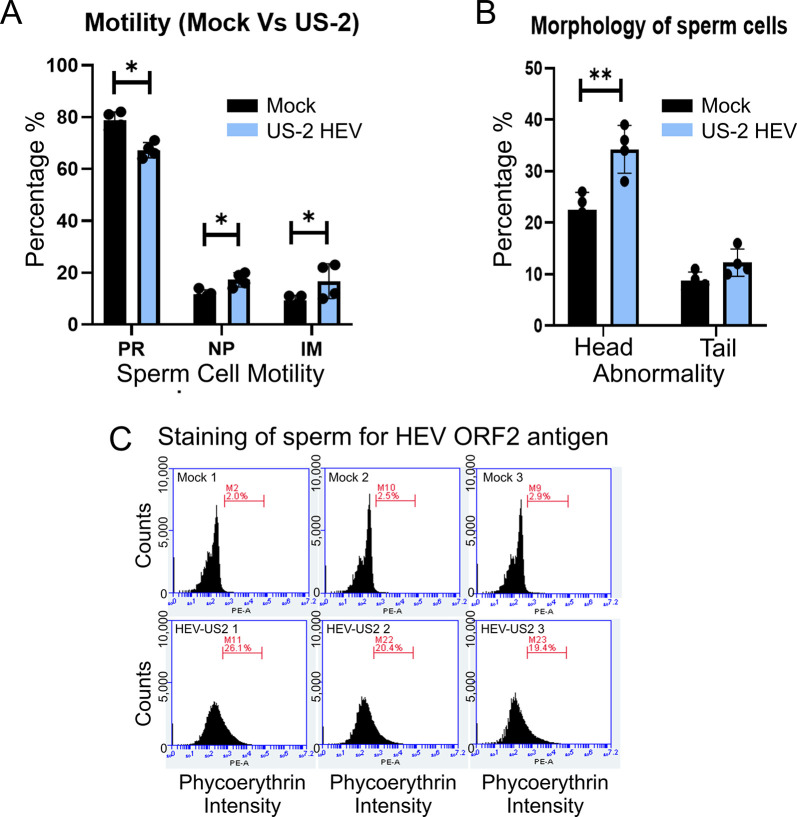
Hepatitis E virus alters spermatozoa motility and morphology. (A) Light microscopic observation of 200 live spermatozoa harvested from mock or infected pig epididymis. Sperm cells demonstrated decreased progressive motility when infected by HEV US-2. PR–progressive motility of sperm (moving active, either linearly or in a circle, regardless of speed); NP–non-progressive motility (all other patterns of motility with absent progression. IM–immobility. (B) Light microscopic observation of live sperm cells harvested from mock or infected pig epididymis. Sperm from US-2 HEV infected pigs showed a significant increase in spermatozoa with head abnormalities. No significant changes were seen in tail of the sperm cells. * = p < 0.05, ** = p < 0.01. (C) Histogram plot was used to demonstrate the data from flow cytometry. Sperm cells from mock non-infected pigs and from US-2 HEV infected pigs.

To evaluate whether HEV infection directly impairs sperm quality, as measured by motility and morphological appearance of sperm cells, semen analysis was immediately performed after collection from the epididymis. Approximately, 200 sperm cells were analyzed to characterize the motility and morphological difference between groups. The progressive sperm motility from US-2 HEV infected sperm (PR% = 64% ± 4%) was found to be significantly lower than the sperm from mock infected (PR% = 78% ± 4%) pigs. The non-progressive sperm motility from US-2 HEV infected sperm (PR% = 18% ± 3%) was found to be significantly higher (p < 0.05) than the sperm from mock infected (PR% = 10% ± 3%) pigs. Immobility of sperm was higher in sperm from the US-2 HEV infected pigs (15% ± 3%) when compared to mock infected (7% ± 2%, p < 0.05) (Fig 3A). Morphologically, we found a higher percentage of abnormal sperm heads (33% ± 4%, p < 0.01) and tails (11% ± 3%) in sperm from US-2 HEV infected pigs than mock infected pigs (Fig 3B). Additionally, flow cytometry analysis of sperm cells quantifies the percentage of sperm cells demonstrating the HEV ORF2 capsid protein (Fig 3C).

## Discussion

HEV continues to present even more complex clinical manifestations due to newly emerging strains of HEV. These manifestations include an increase in extrahepatic and neurological disorders, pregnancy disorders with adverse fetal outcomes, and a lack of approved treatment options beyond ribavirin [4]. The unexpected detection of viral RNA in the semen of infertile men [6] while presenting no viremia and fecal shedding along with our detection of HEV in the spermatozoa potentially shifts the paradigm of host/hepevirus interaction. Our findings place HEV among those viruses which has the potential to be transmitted via sexual intercourse which is troubling considering the high mortality rate associated with HEV infection of pregnant women.

For epidemiological consideration, how HEV genotypes 1 and 2, which are obligate human infecting viruses, persist in the environment has been an ongoing question within the HEV field. There is currently no data for humans harboring the virus in sperm cells, making them a potential extended viral reservoir. Viral persistence in sperm cells in the absence of viremia or fecal shedding is a major concern and could be related to viral tropism for male sexual cells. Currently, we do not know if there are undefined receptors that are common in liver, intestine, ovary, and the sperm head. However, our data suggest that infectious genotype 3 HEV can reside within the sperm head highlighting the need to further characterize its potential ability to persist and transmit sexually.

Further studies are needed to define the mechanisms of the spermatozoa infection, length of persistence in the testis, length of the virus shedding in the spermatozoa, and whether sexual intercourse can result in systemic HEV infection in partners. Given the presence of HEV in sperm cells, future studies will be directed towards understanding the duration of HEV presence in the semen and its concentration. Data on potential ability of HEV to be sexually transmitted will allow public health authorities to recommend safe practices and list HEV on the list of potential pathogens transmitting sexually. HEV gt1, HEV gt2, and HEV gt4 have been described in several human pregnancy mortality cases [1,12]. Despite multiple studies attempting to delineate the mechanism behind pregnancy mortality, a definitive answer is yet to be found. Recent discoveries demonstrating ability of HEV to replicate in the vagina [13] and human male testicular cells [14] demonstrates a new infection scenario that needs detailed investigation and its association with pregnancy mortality. Our study demonstrating infectious HEV in the sperm head raises concerns on a potentially new route of HEV transmission in humans. Sperm related HEV infection should be further explored for its potential to decrease male fertility which has been reported in a 2018 study where HEV RNA was demonstrated in semen samples (3).

Semen alterations were observed, including a decreased progressive motility in US-2 HEV (human HEV) infected pig specimens. These findings suggest a direct viral infection of the sperm cell potentially hinders sperm cell development, in agreement with reported findings [7]. HEV infection has been suggested to impair sperm quality with reductions in sperm motility and vitality in mice via destruction of the blood testis barrier (BTBs) resulting in germ cell necrosis and apoptosis [11]. In addition, HEV infection in mice was demonstrated to damage Leydig cells resulting in spermatogonia necrosis [11]. Another study in non-human primates illustrates the presence of HEV in the spermatogonia with a reduced number of testicular cell populations, primary spermatocytes and Sertoli cells [6]. Thus, we hypothesize that HEV infection impairs the BTB resulting in inflammation that could be a factor resulting in sperm aberrations. In our study, HEV antigen association with the sperm head was performed in fixed sperm cells while morphology and motility were measured in live sperm. We speculate the sperm aberrations observed in sperm from infected pigs is likely due to HEV infection of spermatogonia, inflammation, BTB breakdown, and reduction in Leydig and germ cells. However, the direct association of HEV antigen in the acrosome necessitates an experimental technique that can demonstrate both HEV presence in the sperm head and has the ability to measure sperm quality in live sperm samples. Furthermore, lysates from infected spermatozoa contained infectious HEV capable of infecting HepG2/C3A liver cells. Our study highlights the need to screen human semen samples in geographical regions where endemic HEV causing pregnancy mortality has been reported.

Interestingly, in 2021, differential genomic sequences of HEV were identified in the semen of a chronically infected patient [7]. The antiviral ribavirin can cross the blood-testis barrier to affect viral replication, however the lack of immune clearance may contribute to generation of quasispecies and serve as a source for reinfection. The findings that HEV persists in the semen after detectable viremia and fecal shedding have subsided, suggests that HEV may persist undetected in the testis. Further research is needed to study whether testicular and spermatogonia HEV infection increases the chances of resistant quasispecies in this immune privileged site.

Studies demonstrating the mode of spermatozoa infection and duration of the virus presence in the sperm head leading to the transmission to the ovum are warranted. In addition, this study forms a basis to evaluate the sexual transmission of HEV to pregnant women possibly contributing to pregnancy mortality. Comparative study of HEV receptors in the liver and the sperm head will allow identification of receptors involved in the attachment and the entry of the virus into the sperm cells. Screening of human sperm bank and male pig semen donors needs to be conducted to identify the risk of HEV transmission via artificial insemination. Longitudinal studies to assess the long-term effects of HEV presence in sperm, duration of presence in the sperm including its impact on fertility and reproductive health over time need to be conducted.

Among the merits of our study in terms of translational research of HEV presence in sperm cells, we used a natural HEV host and reservoir pig animal model. Pigs are anatomically and physiologically closer to humans than other small animal models [15]. Our data suggests that the pig model can be used to study HEV-induced sperm health disorders that have been observed in humans. Our findings suggest that HEV infection routes outside of oral and intravenous infection need to be considered for their potential to contribute to higher mortality disease outcomes present in HEV infections when pregnancy is involved and in HEV disease in general.

## Materials and methods

### Ethics statement

All animal experiments in this study were approved by The Ohio State University Institutional Animal Care and Use Committee (IACUC 2020A00000068) and virus studies were approved by the Ohio State Institutional Biosafety Committee (IBC 2016R00000082).

### Collection of semen from US-2 HEV inoculated pigs

Six-week-old conventional pigs were anesthetized and infected via ear vein inoculation with (2 x 10^8^) viral RNA copies (log_10_/ml) of gastrointestinally derived genotype 3 US-2 (human) hepatitis E virus [16]. This route of infection mimics what might occur in the case of human transfusion acquired HEV infection. Testis were collected from eight pigs (US-2 HEV infected, n1 = 4 and mock, n2 = 4) at 84 days post infection (age of pigs = 126 days old). Testis were extracted during necropsy by a veterinarian and stored in a cooled box protected from direct ice contact via a towel. The harvesting of semen samples was done under biosafety level– 2 (BSL2) conditions inside a biosafety cabinet within 8 hours of testis removal. Semen from the epididymis was harvested using a combinatorial approach of aspiration and flushing [17]. The procedure was repeated for both testis from the same animal and the extracted semen was pooled.

### Examination of semen parameters

Semen samples were examined within 15 minutes after collection following the 2010 World Health Organization laboratory manual for the examination of human semen [18]. Motility and morphology of sperm cells between the groups were studied. In brief, 200 spermatozoa per animal sample were examined at 200x magnification. Motility in a sperm cell was studied using three categories: progressive (PR), non-progressive (NP), and immobility (IM). PR refers to a percentage of a sperm’s progressive motility (moving actively, either linearly or in a circle, regardless of speed). NP refers to non-progressive motility (all other patterns of motility with absent progression). IM refers to complete immobility.

### Sperm cell separation from the semen

Semen is a mixture of seminal plasma fluid and sperm cells [19]. Sperm cells were separated using phosphate buffered saline (PBS) in a ratio of 1:10 (volume/volume), and gently mixed to remove the sperm cells clumps. Supernatant was collected and stored at -80°C post centrifugation at 1500 rpm for 10 min. To remove any remnants of seminal plasma, the sperm cell pellet was washed once with 900 μL of PBS, centrifuged, and the supernatant was discarded. The remaining pellet was carefully resuspended with another 900 μL of PBS [20].

### Immunofluorescence staining of sperm cells

To identify hepatitis E virus antigen in sperm cells, 15 μL of the sperm suspension was added in duplicate to Superfrost Plus Slides (Fisherbrand, USA) dried at room temperature and fixed with paraformaldehyde solution (4% in PBS) at 4°C for 5 min, followed by two rounds of washing in PBS for 5 min at room temperature. For the detection of HEV antigens, 30 μL of 1:200 rabbit anti-HEV open reading frame 2 (ORF2) antibody was added to each slide and incubated for 45 minutes at room temperature followed by three washings in PBS for 5 min each. Translation of ORF2 occurs from the subgenomic mRNA. ORF2 protein is synthesized during the later stages of replication and thus used as an indicator of active viral replication [21,22]. Goat anti-rabbit IgG H&L (Alexa Fluor 594; abcam, ab 150080, Cambridge, FL, USA) (1:500) was used as the secondary antibody and the slides were incubated for 30 minutes at room temperature. The slides were then washed three times in PBS for 5 minutes each. 30 μL DAPI (4’,6-diamidino-2-phenylindole) was added as a nuclear counterstain for fluorescence microscopy. Slides were then washed with PBS after two minutes and mounted with fluorescent mounting medium. Slides were imaged using a Keyence microscope at 40x magnification. A sample was considered positive if at least one cell with clear sperm like morphology contained red fluorescence. All specimens were examined in duplicate.

### Flow cytometry analyses of sperm samples

400 μL of the sperm cell suspension was used for flow cytometry quantification of HEV positive cells. Cells were centrifuged at 1500 rpm for 15 minutes and the supernatant was discarded. Cells were then resuspended in 200 μL of 100% cold methanol and left at 4°C for 15 minutes. Cells were centrifuged, resuspended, and washed three times with PBS to remove the methanol. Cells were probed with primary antibody–rabbit anti truncated ORF2 HEV diluted 1:200 in PBS for 30 min at 37°C. Cells were then washed twice with PBS and incubated with secondary antibody–goat anti-rabbit phycoerythrin (Life Technologies, SC 3739) diluted 1:500 in PBS for 30 min at 37°C. Cells were then washed twice in PBS and resuspended in 200 μL of PBS. Fluorescence was analyzed for 100,000 events using a flow cytometer (BD Accuri C6 plus, Biosciences, San Diego, CA, USA). Histogram plots were compared between the infected and mock infected sperm cells.

### Infectivity assay

450 μL of the sperm cell suspension (~4.8 x 10^5^ viral RNA copies) was used to assess the infectiousness of HEV derived from the sperm cells. HepG2/C3A (ATCC HB-8065) human liver cells were used for the study. HepG2/C3A cells were seeded in twelve well plates at 2 x 10^5^ cells per well in 2 ml of DMEM with 10% FBS, penicillin (100 units/ml), streptomycin (100g/ml) and incubated at 37°C for 24 hours. Three repeated freeze and thaw cycles were done with the sperm cell suspension. Cell lysates including both the cell debris and virus were subjected to high-speed centrifugation (10,000 rpm for 5 min) which separates the cell debris. The collected supernatant was used to inoculate the HepG2/C3A liver cells in 12 well plates at 70–80% confluency prior to inoculation. Eight hours post inoculation, the cell culture media was removed, and fresh media was added. Forty-eight hours post inoculation, cells were passed 1:3 and 72 hours later were fixed for immunofluorescence assays. 300 μL supernatant was collected from the wells on day 0, 3, and 5 and replaced with fresh media. RNA extraction was performed followed by RT-qPCR.

### Indirect immunofluorescence

At 5 days post infection, human liver cells were fixed in 100% cold methanol and permeabilized with PBS plus 0.5% tween 20 (PBST). Five percent non-fat dried milk (Sigma-Aldrich, St. Louis, MO, USA) in PBST was used to block non-specific antibody binding. Immunostaining of HEV ORF2 capsid protein was performed using 1:200 rabbit anti-truncated ORF2 HEV. A fluorescently labelled goat anti-rabbit IgG H&L secondary antibody (Alexa Fluor 594; Abcam, Cambridge, FL, USA), 1:500, was used. DAPI was used to stain the nucleus. For quantification of virus infectivity, wells were manually observed for specific fluorescence.

### Flow cytometry analyses of HepG2/C3A cells

Five days post infection, HepG2/C3A cells were trypsinized and pelleted. Cells were then fixed in 200 μL of 100% methanol at 4°C. After overnight storage at -80°C, cells were centrifuged out of methanol, washed, and resuspended in phosphate buffered saline (PBS). Cells were blocked-in blocking solution (5% non-fat dried milk, 0.1% Triton X-100 in PBS; PBST) in a 96-well plate for 30 min at 37°C. Cells were probed with primary antibody–rabbit anti truncated ORF2 HEV diluted 1:200 in PBS for 30 min at 37°C. Cells were then washed twice with PBS and incubated with secondary antibody–goat anti-rabbit phycoerythrin (Life Technologies, SC 3739) diluted 1:500 in PBS for 30 min at 37°C. Cells were then washed twice in PBS and resuspended in 200 μL of PBS. Fluorescence was analyzed for 100,000 events using a flow cytometer (BD Accuri C6 plus, Biosciences, San Diego, CA, USA). Gates were set to exclude dead cells, doublet discrimination based on forward and side scatter profiles, and mock infected cells were used to gate background fluorescence [22].

### RNA extraction and RT-qPCR

RNA extraction was performed using TRIzol reagent (Invitrogen). Extracted RNA from serum, rectal swabs, sperm cells, seminal fluid and homogenized tissues were subjected to reverse transcriptase quantitative polymerase chain reaction (RT-qPCR). A forward primer US-2 HEV F, 5′-GGTGGTTTCTGGGGTGAC-3′, a reverse primer US-2 HEV R, 5′-AGGGGTTGGTTGGATGAA-3′, and a probe 5’-FAM-TGATTCTCAGCCCTTCGC-Dabcyl-3’ were used for the detection of US-2 HEV. A 10-fold serial dilution of the capped US-2 HEV RNA (10^7^ to 10^1^ copies) was used as the standard for the quantification of the viral genome copy numbers.

### Statistical analysis

All quantitative data are presented with the mean and standard deviation. Analyses of independent data were performed by Student’s unpaired two-tailed t test and two-way ANOVA followed by post-hoc test using GraphPad Prism 9.4.1. with p < 0.05 considered as statistically significant.

## Supporting information

S1 Data Data that underlies this paper. (XLSX)
